# Exploring *Leptospira interrogans* FDAARGOS_203: Insights into AMR and Anti-Phage Defense

**DOI:** 10.3390/microorganisms12030546

**Published:** 2024-03-08

**Authors:** Pavlo Petakh, Valentyn Oksenych, Oleksandr Kamyshnyi

**Affiliations:** 1 Department of Biochemistry and Pharmacology, Uzhhorod National University, 88000 Uzhhorod, Ukraine; pavlo.petakh@uzhnu.edu.ua; 2 Department of Microbiology, Virology and Immunology, I. Horbachevsky Ternopil National Medical University, 46001 Ternopil, Ukraine; 3 Broegelmann Research Laboratory, Department of Clinical Science, University of Bergen, 5020 Bergen, Norway

**Keywords:** leptophages, Cas system, bacteriophages, *Leptospira interrogans*, antibiotic

## Abstract

Leptospira, which are known to be important disease-causing agents transmitted between animals and humans, result in significant illness and, in some cases, significant death in human populations. This purpose of this study was to examine the genomic structure of *Leptospira interrogans* serovar Copenhageni strain FDAARGOS_203 to identify the specific genetic factors that contribute to antimicrobial resistance (AMR) and defense against phages. The genome, consisting of two contigs totaling 4,630,574 base pairs, underwent thorough examination for protein-coding sequences, transfer RNA genes, and ribosomal RNA genes. A total of twenty-two antibiotic resistance genes that specifically target essential cellular processes such as cell wall synthesis, DNA replication, and protein synthesis have been identified. Significant among these were *gidB*, *gdpD*, and *ggsA*, each involved in separate aspects of antibiotic resistance. In addition, the investigation explored the defense mechanisms of bacteriophages, revealing the presence of defense islands that contain a range of anti-phage systems, including RM_Type_IV, PrrC, Borvo, CAS_Class1-Subtype-IC, and CAS_Class1-Subtype-IB. This comprehensive genomic analysis enhances our understanding of the molecular mechanisms that determine Leptospira’s ability to adapt to various environments. The identified genetic factors linked to AMR and defense against phages not only enhance our scientific comprehension, but also provide a basis for focused interventions to reduce the impact of leptospirosis.

## 1. Introduction

Leptospira, major agents of zoonotic disease, cause considerable morbidity and, in some instances, significant mortality in humans [1,2,3,4,5,6,7,8,9]. The genus *Leptospira* comprises over 20 species based on DNA relatedness, with more than 350 serovars identified based on surface agglutinating lipopolysaccharide antigens [10]. These species are broadly categorized into three groups. Saprophytic species like *Leptospira biflexa* are not associated with disease. Pathogenic species such as *Leptospira interrogans* and *Leptospira borgpetersenii* cause leptospirosis globally, ranging from mild or asymptomatic infection to severe forms resulting in multiple organ failure and death. An intermediate group, including *Leptospira fainei* and *Leptospira licerasiae*, may be associated with infection and mild disease.

Despite the clinical significance of leptospirosis, there is a notable lack of comprehensive data regarding the protective mechanisms employed by leptospires against antibiotics and phages. *Leptospira* spp. exhibit intrinsic resistance to various antimicrobial agents, though the specific mechanisms responsible remain unidentified [11,12]. Nevertheless, resistance to sulfonamides, neomycin, actidione, polymyxin, nalidixic acid, vancomycin, and rifampicin has facilitated the development of selective media for isolating leptospires [13].

Current recommendations for treating human leptospirosis involve penicillin, ampicillin, ceftriaxone, or cefotaxime [1,14]. Alternatives, particularly for those with allergies or in non-hospital settings, include oral doxycycline or azithromycin. In veterinary settings, a penicillin–streptomycin combination is the preferred therapy for acute leptospirosis, although ampicillin, amoxicillin, tetracyclines, tulathromycin, and third-generation cephalosporins have also been utilized [15]. Tilmicosin presents an additional alternative [16].

Renewed interest in bacteriophages as alternatives to antibiotics and their role in bacterial evolution has emerged, yet little is known about phage diversity within the *Leptospira* genus [17,18]. As far as our knowledge extends, the only phages identified, purified, and characterized within the *Leptospira* genus are vB_LbiM_LE1 (also known as LE1), vB_LbiM_LE3 (LE3), and vB_LbiM_LE4 (LE4) [19,20]. A quest for prophages closely associated with LE4 in Leptospira genomes led to the discovery of a corresponding plasmid in *L. interrogans* and a prophage-like region in the preliminary genome of a clinical strain of *L. mayottensis*. Through long-read whole-genome sequencing of *L. mayottensis*, it was discovered that the genome harbored a circular plasmid resembling the LE4 phage [21].

Saint Girons et al. first isolated bacteriophages from *Leptospira* species in 1990, but their exploration remains limited [20]. Schiettekatte et al. demonstrated that leptophages utilize lipopolysaccharides (LPS) as receptors on bacterial cells [18]. Bacteria engage in a continuous arms race, evolving defense mechanisms against the expanding arsenal of phage weapons [22]. These defense systems, discovered in recent years, protect against phages through various molecular mechanisms. Anti-phage defense systems exhibit a non-random distribution in microbial genomes, often forming “defense islands” where multiple systems cluster together [23,24,25].

The strain FDAARGOS_203, being a reference strain, provides a unique opportunity to explore the genetic basis of antibiotic and phage resistance in *Leptospira interrogans*. Through a comprehensive examination of the genome, we aim to contribute valuable insights into the genetic factors governing AMR and anti-phage defense, enhancing our understanding of leptospirosis and paving the way for more effective therapeutic interventions.

## 2. Materials and Methods

### 2.1. Data

The genome of *Leptospira interrogans* serovar Copenhageni strain FDAARGOS_203 was downloaded in FASTA format files from the Bacterial and Viral Bioinformatics Resource Center (BV-BRC) database (GenBank: GCA_002073495.2) [26]. The genome assembly was conducted using the NCBI RefSeq assembly with the identifier GCF_002073495.2. The assembly was submitted by the University of Maryland School of Medicine Institute for Genome Sciences (IGS)—sequencing center. The assembly method employed was HGAP v. 3, utilizing PacBio and Illumina sequencing technologies [26]. The leptospiral genome was annotated using the RAST tool kit (RASTtk) [27].

### 2.2. Detection of AMR Genes

The genomes were then analyzed using the PATRIC tool from the BV-BRC to identify antimicrobial resistance genes [28]. The Genome Annotation Service in PATRIC uses the k-mer-based AMR gene detection method, which utilizes PATRIC’s curated collection of representative AMR gene sequence variants and assigns to each AMR gene a functional annotation and a broad mechanism of antibiotic resistance.

### 2.3. Detection of Antiviral Systems

DefenseFinder was used to identify anti-phage defense systems [29]. DefenseFinder utilizes MacSyFinder27, a program dedicated to the detection of macromolecular systems, functioning with one model per system [30]. This approach involves a two-step process: first, the detection of all proteins involved in a macromolecular system through a homology search using Hidden Markov Model (HMM) profiles; second, the application of decision rules to retain only the HMM hits that satisfy the genetic architecture of the system of interest. Genomic features such as phage and genomic island sequences were recognized using online bioinformatic tools such as Island Viewer [31].

### 2.4. Phylogenetic Analysis

The genome sequence data were uploaded to the Type (Strain) Genome Server (TYGS), a free bioinformatics platform available under https://tygs.dsmz.de (accessed on 29 February 2024), for a whole-genome-based taxonomic analysis [32]. The analysis also made use of recently introduced methodological updates and features [33]. Information on nomenclature, synonymy, and associated taxonomic literature was provided by TYGS’s sister database, the List of Prokaryotic names with Standing in Nomenclature (LPSN, available at https://lpsn.dsmz.de (accessed on 29 February 2024)) [33]. The TYGS analysis was subdivided into the following steps.

#### 2.4.1. Determination of Closely Related Type Strains

Determination of the closest type strain genomes was performed in two complementary ways: First, all user genomes were compared against all type strain genomes available in the TYGS database via the MASH algorithm, a fast approximation of intergenomic relatedness, and, the ten type strains with the smallest MASH distances chosen per user genome [34]. Second, an additional set of ten closely related type strains was determined via the 16S rDNA gene sequences. These were extracted from the user genomes using RNAmmer and each sequence was subsequently BLASTed against the 16S rDNA gene sequence of each of the currently 20,415 type strains available in the TYGS database [35,36]. This was used as a proxy to find the best 50 matching type strains (according to the bitscore) for each user genome and to subsequently calculate precise distances using the Genome BLAST Distance Phylogeny approach (GBDP) under the algorithm ‘coverage’ and distance formula d5. These distances were finally used to determine the 10 closest type strain genomes for each of the user genomes.

#### 2.4.2. Pairwise Comparison of Genome Sequences

For the phylogenomic inference, all pairwise comparisons among the set of genomes were conducted using GBDP and accurate intergenomic distances inferred under the algorithm ‘trimming’ and distance formula d5 [37]. A total of 100 distance replicates were calculated for each. Digital DDH values and confidence intervals were calculated using the recommended settings of the GGDC 4.0 [37].

#### 2.4.3. Phylogenetic Inference

The resulting intergenomic distances were used to infer a balanced minimum evolution tree with branch support via FASTME 2.1.6.1 including SPR postprocessing [38]. Branch support was inferred from 100 pseudo-bootstrap replicates each. The trees were rooted at the midpoint and visualized with PhyD3 [39].

### 2.5. Figures and Statistical Analysis

Statistical analysis and visualization were performed using SRplot and jvenn [40,41].

## 3. Results

### 3.1. Genome Assembly and Annotation

The genome of Leptospira interrogans serovar Copenhageni strain FDAARGOS_203 was assembled using the HGAP v. 3 method at the University of Maryland School of Medicine Institute for Genome Sciences (IGS)—sequencing center (NCBI RefSeq assembly GCF_002073495.2), and we conducted an analysis of its genetic content. The assembly consisted of two contigs, totaling 4,630,574 base pairs, with an average G+C content of 35.05% (Table 1).

Quality control measures, such as the removal of low-quality reads and the trimming of adapters, were performed prior to assembly. The genome was then annotated using the RAST toolkit (RASTtk) and assigned a unique genome identifier of 173,581. The genome contained 4479 protein-coding sequences (CDSs), 37 transfer RNA (tRNA) genes, and 3 ribosomal RNA (rRNA) genes. The annotation revealed 2305 hypothetical proteins and 2174 proteins with functional assignments (Table 2).

The proteins with functional assignments included 671 proteins with Enzyme Commission (EC) numbers, 556 with Gene Ontology (GO) assignments, and 517 proteins that were mapped to KEGG pathways. PATRIC annotation includes two types of protein families, and this genome has 4061 proteins that belong to the genus-specific protein families (PLFams) and 4160 proteins that belong to the cross-genus protein families (PGFams) (Table 3) [42,43,44,45].

**Figure 1:**
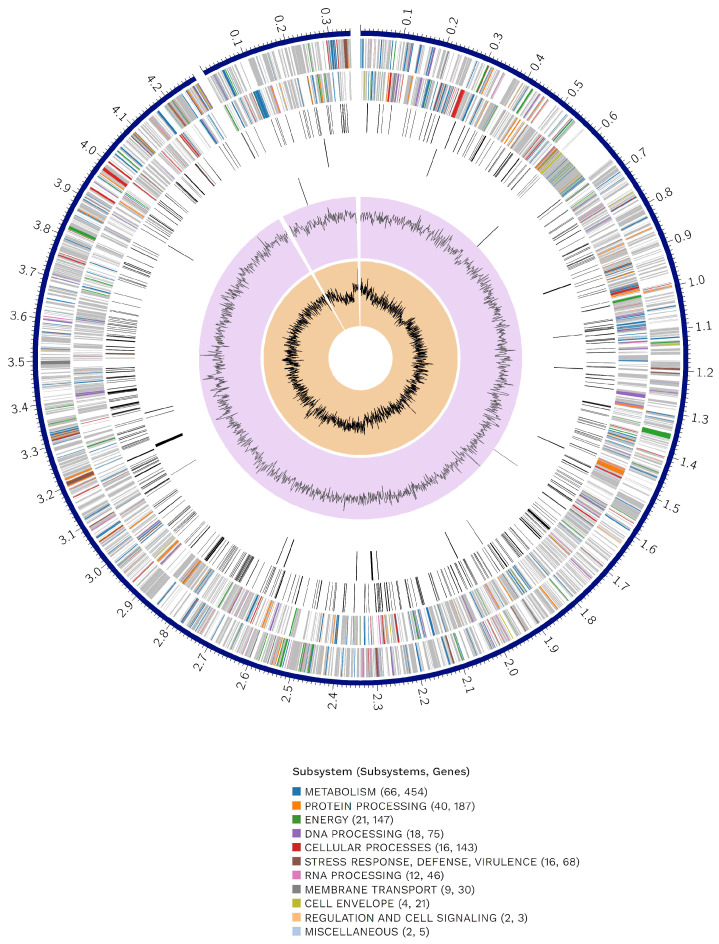
Circular genome display. From outer to inner rings, the contigs, CDSs on the forward strand, CDSs on the reverse strand, RNA genes, CDSs with homology to known antimicrobial resistance genes, CDSs with homology to known virulence factors, GC content, and GC skew. The colors of the CDSs on the forward and reverse strands indicate the subsystems to which these genes belong. The cut-out section in the circle of the genome display represents the plasmid genome. The complete genome of the strain includes 1 chromosome (CP020414.2) with a length of 4,280,582 bp and a plasmid (CP020413.2) of 350,181 bp.

A circular graphical representation displays the genome annotations, including contigs, CDSs on the forward and reverse strands, RNA genes, and features related to antimicrobial resistance and virulence factors (Figure 1).

**Figure 2:**
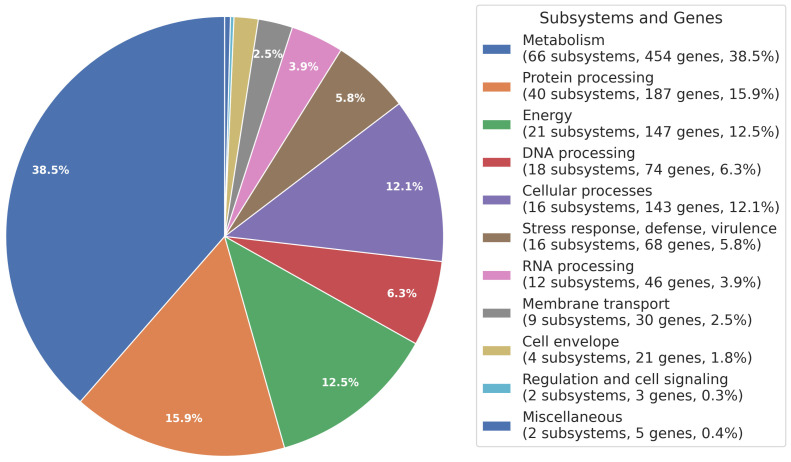
Subsystem overview. The distribution of subsystems, molecular pathways, and processes is indicated by a color code and presented as percentages on a pie chart.

The distribution of subsystems in this genome was illustrated, providing an overview of its functional organization (Figure 2).

### 3.2. Specialty Genes

Several genes annotated in the genome demonstrated homology to known transporters, virulence factors, drug targets, and antibiotic resistance genes. Specifically, 22 antibiotic resistance genes were identified using the PATRIC database, along with one drug target and 67 transporter genes (Table 4). The antibiotic resistance genes targeted various essential cellular functions, such as cell wall synthesis, DNA replication, and protein synthesis (Table 4).

### 3.3. Phylogenetic Analysis

**Figure 3:**
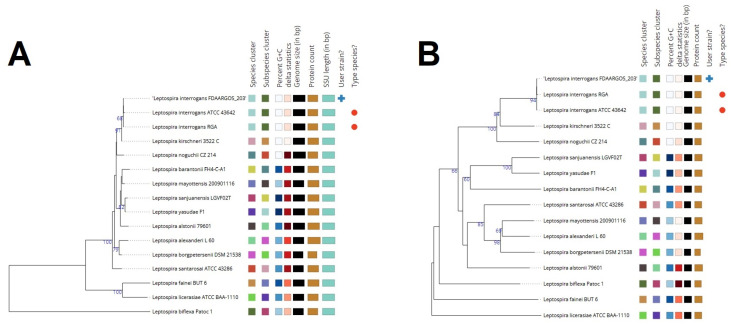
(**A**). GBDP tree (16S rDNA gene sequence-based). The branch lengths are scaled in terms of GBDP distance formula d5. The numbers above branches are GBDP pseudo-bootstrap support values > 60% from 100 replications, with an average branch support of 59.2%. The tree was rooted at the midpoint. (**B**). GBDP tree (whole-genome sequence-based). The branch lengths are scaled in terms of GBDP distance formula d5. The numbers above branches are GBDP pseudo-bootstrap support values > 60% from 100 replications, with an average branch support of 70.9%. The tree was rooted at the midpoint.

The phylogenetic placement of the Leptospira interrogans serovar Copenhageni strain FDAARGOS_203 genome was determined using reference and representative genomes. The analysis, conducted with RaxML and fast bootstrapping, identified closely related genomes based on Mash/MinHash comparisons. The resulting tree (Figure 3) provides insights into the evolutionary relationships of this strain within the broader context of Leptospira species.

### 3.4. Anti-Phage Systems

The genome analysis also revealed the presence of various anti-phage defense systems, such as RM_Type_IV, PrrC, Borvo, CAS_Class1-Subtype-IC, and CAS_Class1-Subtype-IB. These defense mechanisms likely play a crucial role in protecting the bacterium from phage attacks and contribute to its survival in various environments.

### 3.5. AMR-Associated Genes

A study of the genome of the Leptospira interrogans serovar Copenhageni strain FDAARGOS_203 revealed a cluster of genes associated with antibiotic resistance. These genes impact essential cellular functions, including protein synthesis, DNA replication, and cell wall synthesis. Notably, the gidB gene was identified, suggesting its involvement in conferring resistance through absence. Additionally, the gdpD and pgsA genes were found to be associated with altering cell wall charge, contributing to antibiotic resistance (Table 5).

## 4. Discussion

Our study was conducted to analyze, for the first time, the genome of a reference strain of *Leptospira* for the presence of anti-phage systems and mechanisms of resistance to antibiotics. This study provides a solid foundation for initiating new research in this field.

**Figure 4:**
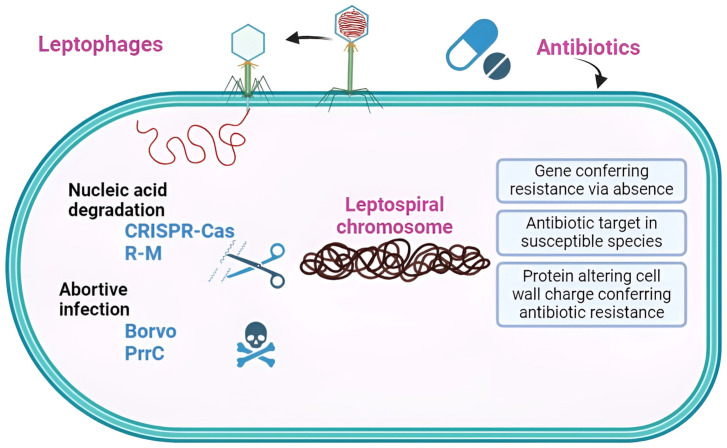
Schematic representation of AMR and anti-phage systems in *Leptospira interrogans* FDAARGOS_203. This strain possesses four anti-phage defense systems and three AMR mechanisms.

We identified only two studies that investigated *Leptospira* anti-phage systems, both of which focused solely on Clustered Regularly Interspaced Short Palindromic Repeats (CRISPRs) and their subtypes [46,47]. CRISPR Types I and III are considered dominant for *Leptospira*. CRISPR-Cas systems exhibit variability, consisting of six types (I–VI) across two classes, totaling 50 subtypes based on their sequences [48]. Despite this diversity, two fundamental functions are conserved among the different types [49]. The first involves cas genes, which encode Cas proteins responsible for manipulating nucleic acids. The second entails a noncoding DNA sequence array featuring a short, partially palindromic, repetitive sequence interspersed with variable sequences (spacers) that dictate the targets. Functioning as adaptive immunity, the system’s adaptability is evident in acquiring spacers from invaders. These acquired spacers can undergo transcription to form small CRISPR RNAs (crRNAs) that, when combined with Cas nucleases, serve as a defense mechanism against foreign nucleic acids [49]. However, our discovery revealed additional methods of protection against leptophages, specifically, RM_Type_IV (also known as the type IV restriction–modification (R–M) system), PrrC, and Borvo (Figure 4).

The detection of the Type IV restriction–modification system is particularly interesting. R–M systems, the most studied class of defense systems since their discovery in the 1960s [50]. The R–M systems are categorized into four types. Types I–III include methyltransferases that methylate the host DNA and corresponding restriction endonucleases (RNases) that cleave invasive and unmethylated DNA [49]. In contrast, the type IV R–M system lacks methyltransferases and instead specifically recognizes methylated DNA. This mechanism may act against phages attempting to evade defense systems through modified genomes [51]. The finding of Type IV requires further research.

If the R–M system is compromised by a phage inhibitor as the primary defense, PrrC can still provide a secondary line of defense [52]. The effector protein PrrC functions to complement an R–M system by cleaving tRNALys within the anticodon loop, located upstream of the wobble nucleotide. This action leads to a halt in phage protein synthesis and inhibits phage growth. PrrC acts as a protector of EcoprrI’s activity, which can be rendered inactive by the Stp peptide from phage T4 during the initial stages of infection. The inactivation of EcoprrI by Stp triggers a structural change that activates PrrC. Consequently, PrrC releases its nuclease activity, causing a cessation in both host and phage growth. Due to its interference with the host’s translation machinery, PrrC is classified as an abortive infection system [53,54,55,56].

Borvo is a single-gene anti-phage system that was identified through bioinformatic prediction and experimental validation. Mutations in the phage DNA polymerase can allow phages to escape Borvo defense, indicating that it could be the trigger of the system. Borvo is a suspected abortive infection. However, as far as we are aware, the precise molecular mechanism of Borvo is unknown [57,58]. Among the 22,803 complete genomes of RefSeq, Borvo is detected in 177 genomes (0.78%). The system was detected in 79 different species [26]. While this antiphage system is relatively rare, we were able to detect it in our strain.

Leptospires have evolved several defense mechanisms against bacteriophages, and CRISPR is just one of them. Our findings make a significant contribution to future research, particularly for the development of potential drugs for treating leptospirosis in animals or humans.

Additionally, we identified 20 genes responsible for leptospirosis resistance to antibiotics. The apparent absence of significant antimicrobial resistance emergence in *Leptospira* raises the question of why this has not occurred. Leptospiral infections are typically monomicrobial, limiting opportunities for horizontal resistance gene acquisition. Moreover, there is no experimental evidence of foreign DNA uptake by *Leptospira* spp., although genomic analyses support this notion [59]. Finally, human leptospirosis is a dead-end infection, with human-to-human transmission being extremely rare.

We have identified three modes of AMR in our strain. The first mode involves the antibiotic target in susceptible species, which is defined as antibiotic-sensitive wild-type bacterial components. However, mutations may occur, rendering them not susceptible. The second mode involves protein alteration leading to cell wall charge changes that confer antibiotic resistance through cell wall modification. The last mode is the gene conferring resistance via absence, defined as the deletion of a gene or its product resulting in resistance. For instance, the deletion of a porin gene blocks the drug from entering the cell [60]. Alterations in the target sites of antibiotics may therefore be the major cause of antibiotic resistance in *Leptospira* [61]. Experimental evidence demonstrates that the in vitro selection process can lead to the emergence of resistance to spectinomycin and streptomycin in Leptospira. This resistance is attributed to spontaneous mutations occurring in the target genes 16S rRNA and rpsL, respectively [62,63].

## 5. Conclusions

In summary, our analysis of *Leptospira interrogans* strain FDAARGOS_203′s genome unveiled four anti-phage defense systems—CRISPR-Cas, RM, PrrC, and Borvo. Particularly interesting was the discovery of the Borvo system, considered quite rare with an unexplained mechanism to date.

The genome analysis also revealed 22 antibiotic resistance genes, which may explain resistance to certain types of antibiotics, such as fluoroquinolones. However, it is noteworthy that resistance to antibiotics is not generally characteristic of leptospiras.

In conclusion, our research contributes valuable insights into the genetic basis of antibiotic resistance and anti-phage defense in Leptospira, setting the stage for further exploration and the development of innovative therapeutic strategies.

## Figures and Tables

**Table 1:** Assembly details.

Feature	Value
Contigs	2
GC Content	35.05
Plasmids	1
Contig L50	1
Genome Length	4,630,574 bp
Contig N50	4,280,403
Chromosomes	1

**Table 2:** Annotated genome features.

Feature	Value
CDS	4479
Repeat Regions	485
tRNA	37
rRNA	3

**Table 3:** Protein features.

Feature	Value
Hypothetical proteins	2305
Proteins with functional assignments	2174
Proteins with EC number assignments	671
Proteins with GO assignments	556
Proteins with pathway assignments	517
Proteins with PATRIC genus-specific family (PLfam) assignments	4061
Proteins with PATRIC cross-genus family (PGfam) assignments	4160

**Table 4:** Specialty genes.

Type	Source	Genes
Antibiotic Resistance	PATRIC	22
Drug Target	DrugBank	1
Transporter	TCDB	67

**Table 5:** Genes associated with antimicrobial resistance.

Type	Gene Names
Antibiotic target in susceptible species	*alr*, *ddl*, *dxr*, *ef-g*, *ef-tu*, *fola*, *dfr*, *folp*, *gyra*, *gyrb*, *iso-tRNA*, *mura*, *rho*, *rpoB*, *rpoC*, *s10p*, *s12p*
Gene conferring resistance via absence	*gidB*
Protein altering cell wall charge conferring antibiotic resistance	*gdpD*, *pgsA*

## Data Availability

The data that support the findings of this study are available on request from the corresponding author.
